# Antibiotic resistance rates in *Cutibacterium acnes* isolated from patients with acne vulgaris: a systematic review and meta-analysis

**DOI:** 10.3389/fmicb.2025.1565111

**Published:** 2025-06-04

**Authors:** Chunxiao Zhu, Baozhen Wei, Yang Li, Changyuan Wang

**Affiliations:** ^1^School of Clinical Medicine, Shandong Second Medical University, Weifang, China; ^2^Department of Dermatology, Qingdao Hospital, University of Health and Rehabilitation Sciences (Qingdao Municipal Hospital), Qingdao, China

**Keywords:** acne vulgaris, *Cutibacterium acnes*, antibiotic resistance, prevalence, meta-analysis

## Abstract

**Background:**

*Cutibacterium acnes* (*C. acnes*) is closely related to the pathogenesis of acne, and studies related to the antibiotic resistance rates of *C. acnes* have been reported worldwide; however, relevant systematic reviews and meta-analyses are still lacking. The aim of this study was to systematically evaluate the resistance in *C. acnes* to relevant antibiotics, that this information may be used to provide a rational basis for the antibiotic treatment of acne.

**Methods:**

Relevant studies in PubMed, the Cochrane Library, EMBASE, Web of Science, China National Knowledge Infrastructure (CNKI) and Wanfang Data were systematically searched from January 1, 2005, to April 1, 2025, and the resistance rates of *C. acnes* isolates to quinolones, macrolides, tetracyclines, and other relevant antibiotics were collected. The combined resistance rate was calculated via the *R* language program package 4.3.2, with subgroup analyses based on different years, continents, countries, provinces in China and different drug susceptibility testing methods.

**Results:**

A total of 8,846 studies were systematically retrieved and 23 studies were included, corresponding to 2,046 isolates of *C. acnes*, which have shown antibiotic resistance rates ranging from high to low: 48.17% (95% CI: 41.16–55.24%) for roxithromycin, 45.64% (95% CI: 20.49–73.22%) for clarithromycin, 43.33% (95% CI: 27.81–60.29%) for azithromycin, 29.20% (95% CI: 22.14–37.43%) for erythromycin, 22.38% (95% CI: 14.69–32.56%) for clindamycin, 5.93% (95% CI: 2.91–11.69%) for levofloxacin, 2.44% (95% CI: 0.99–5.89%) for doxycycline, 1.47% (95% CI: 0.00–85.72%) for trimethoprim-sulfamethoxazole (TMP–SMX), 1.31% (95% CI: 0.45–3.70%) for tetracycline, 0.28% (95% CI: 0.04–1.94%) for chloramphenicol, 0.22% (95% CI: 0.03–1.89%) for minocycline. Subgroup analysis revealed that, compared with those in other regions, the resistance rates to macrolides and clindamycin were higher in China. In addition, the levofloxacin, erythromycin, and clindamycin resistance rates were progressively increasing over time.

**Conclusion:**

In certain regions, the relatively high antibiotic resistance rates (e.g., 77% (95% CI: 62–87%) for clarithromycin in China) in *C. acnes* isolates may be attributed to the overuse of antibiotics in acne treatment. The resistance rates in *C. acnes* to tetracyclines, such as 2.44% (95% CI: 0.99–5.89%) for doxycycline, remain relatively low, which allows tetracyclines to continue serving as first-line antibiotics for acne treatment. In addition, the resistance rates to levofloxacin, erythromycin, and clindamycin markedly increased over time (*p* < 0.05). This emphasizes the significance of rational use of the antibiotics in acne treatment.

## Introduction

Acne is a chronic inflammatory skin disease involving hair follicles and sebaceous glands. The main sites of acne are the face, chest and back, where papules, nodules, and pustules appear, and some patients also experience scarring (Xu and Li, 2019). Owing to its long duration, easy recurrence and the possibility of severe scarring, acne seriously affects the physical and mental health of patients, and some patients may suffer from anxiety, depression or even suicidal tendencies (Samuels et al., 2020).

The exact etiology and pathogenesis of acne are still unclear, and current studies have shown that it is associated mainly with hormone levels, increased sebum secretion, abnormal keratinization of follicular sebaceous gland ducts, and microbial colonization (Habeshian and Cohen, 2020). *Cutibacterium acnes* (*C. acnes*) plays an integral role in the pathogenesis of acne and is considered the most dominant flora within the follicular sebaceous glands of acne patients (Dreno et al., 2018; Platsidaki and Dessinioti, 2018). *C. acnes* belongs to the genus *Cutibacterium*, which currently comprises five recognized species: *C. acnes*, *Cutibacterium avidum* (*C. avidum*), *Cutibacterium granulosum* (*C. granulosum*), *Cutibacterium modestum* (*C. modestum*), and *Cutibacterium namnetense* (*C. namnetense*) (Dekio et al., 2021). As the most prevalent species, *C. acnes* is associated not only with acne but also with various deep-seated infections, including prosthetic joint infections (PJIs) (Kusejko et al., 2021) and pulp infections (Alvarez-Munoz et al., 2025). Additionally, *C. acnes* has been implicated in SAPHO syndrome (Corbisiero et al., 2023). *C. avidum* predominantly colonizes moist skin areas and it linked to implant-associated infections, such as PJIs and gluteal implant infections (Boni et al., 2018; Isabel Cristina and Diego, 2025). The clinical isolation rate of *C. granulosum* is relatively low, with associations primarily noted in surgical site infections and skin and soft tissue infections (Broly et al., 2020). *C. modestum* is a recently identified species, with its first clinically documented case associated with vertebral osteomyelitis (Koyama et al., 2022). *C. namnetense,* a novel species, remains clinically understudied but has been reported in liver abscesses (Yasutomi et al., 2021). *C. acnes* contributes to disease progression through multiple mechanisms. It activates keratinocytes and monocytes through toll-like receptors (TLRs), triggering the release of pro-inflammatory factors, including IL-1β, IL-8, IL-12, and TNF-α, which drive perifollicular inflammation (Dreno et al., 2020). Additionally, *C. acnes* promotes the differentiation of CD4 + naive T cells into T helper (Th)17 cells, promoting IL-17 secretion and exacerbating hair follicle inflammation and abnormal keratinization (Mias et al., 2023). Christy-Atkins-Munch-Petersen factors, encoded in the *C. acnes* genome, function as membrane-forming pore toxins and host tissue-degrading enzymes. These secreted proteins exhibit cytotoxic effects on keratinocytes and macrophages, potentially amplifying skin inflammation (Achermann et al., 2014). Biofilm formation further enhances *C. acnes* pathogenicity. It can establish biofilms within hair follicles, reducing antimicrobial susceptibility *via* physical barriers and fostering chronic infection (Poudel et al., 2021; Ruffier D'Epenoux et al., 2024).

Antimicrobial therapies for *C. acnes* mainly include topical erythromycin and clindamycin, as well as oral tetracyclines (Adler et al., 2017). Due to the high prevalence of acne, the widespread use of antibiotics and the long course of the disease, a gradual increase in the resistance rates of relevant antibiotics against *C. acnes* has been observed (Karadag et al., 2021). Some *C. acnes* isolates exhibited cross-resistance to macrolide-lincosamide (ML), macrolide-lincosamide-streptomycin (MLS), or tetracyclines and relatively high minimum inhibitory concentration (MIC) (Abdel Fattah and Darwish, 2013; Sheffer-Levi et al., 2020). Although meta-analyses of the resistance rate of *C. acnes* have been conducted (Alvarez-Sanchez et al., 2016; Beig et al., 2024), however, considerable time has passed since the publication of these studies, and there are some differences in the types of antibiotics studied and subgroup analyses. Therefore, the primary objective of this study was to assess the antibiotic resistance rates in *C. acnes* in a timely manner, as well as the spatial and temporal variations in resistance rates, thereby promoting more rational antibiotic use in acne treatment.

## Methods

### Search strategy

By searching PubMed, the Cochrane Library, EMBASE, Web of Science, China National Knowledge Infrastructure (CNKI) and Wanfang Data, studies on antibiotic resistance in *C. acnes* from January 1, 2005, to April 1, 2025, the types of studies included were cross-sectional studies. The specific keywords used for the search are shown in Supplementary Table S1, with a focus on the title/abstract/keyword fields. The study protocol was registered with the Prospective Register of Systematic Reviews (https://www.crd.york.ac.uk/PROSPERO/) (ID: CRD42024618176), and systematic evaluation and meta-analysis were performed according to the Preferred Reporting Items for Systematic Reviews and Meta-Analyses (PRISMA) guidelines (Shamseer et al., 2015).

### Inclusion and exclusion criteria

The following criteria were included in the included studies: (1) The subject of these studies was the resistance rate of *C. acnes*. (2) The bacterial samples were from skin acne isolates. (3) Complete data from the studies are provided (including first author, sample collection time, region, number of isolated strains, experimental methodology, resistance criteria and number of antibiotic-resistant strains isolated). (4) The studies are original. (5) Replicates are selected from the most recently published studies. (6) High-quality studies with a score of 4 or more according to the Joanna Briggs Institute (JBI) tailored tool for epidemiology (Munn et al., 2015) and its adapted version (Savoldi et al., 2018). (7) The resistance criteria are required to be adopted as the criteria specified for the Clinical and Laboratory Standards Institute (CLSI) (Clinical and Laboratory Standards Institute Performance Standards for Antimicrobial Susceptibility Testing, 2018), which is known as the National Committee for Clinical Laboratory Standards (NCCLS) (National Committee for Clinical Laboratory Standards Methods for Antimicrobial Susceptibility Testing of Anaerobic Bacteria, 2004) until 2005 or the European Committee on Antimicrobial Susceptibility Testing (EUCAST) (European Committee on Antimicrobial Susceptibility Testing Breakpoint Tables for Interpretation of MICs and Zone Diameters, 2019). (8) Drug susceptibility tests include the broth microdilution method, the agar dilution method, or gradient tests (E-test, spiral gradient test).

The exclusion criteria were as follows: (1) Samples from unknown sources as well as non-dermal samples. (2) Studies providing incomplete data. (3) Reviews, meta-analyses, case reports, and commentaries. (4) Studies with low quality assessment scores. (5) Ambiguous resistance criteria as well as nonCLSI or EUCAST criteria. (6) Studies employing the disk diffusion method.

### Data extraction and quality assessment

Studies were extracted independently by two authors (C-XZ and B-ZW) and screened according to the inclusion and exclusion criteria. Cohen Kappa coefficient was used to evaluate the consistency of the studies extracted by the two authors (C-XZ and B-ZW). Cohen proposed that the kappa coefficient be interpreted as follows: values ≤ 0 indicate no agreement, 0.01–0.20 indicate no to slight agreement, 0.21–0.40 indicate fair agreement, 0.41–0.60 indicate moderate agreement, 0.61–0.80 indicate substantial agreement, and 0.81–1.00 indicate almost perfect agreement (McHugh, 2012). When there were differences of opinion, they were resolved through discussion or with the assistance of a third investigator (Y L). The following data were extracted from the articles: first author, year of publication, duration of the experiment, region, number of patients, number of *C. acnes* strains, resistance rates to different antibiotics, resistance criteria, experimental methods, other factors that may be associated with resistance rates, and other important results.

The included studies were independently assessed by two authors (C-XZ and B-ZW) via the JBI tailored tool for epidemiology (Munn et al., 2015) and its adapted version (Savoldi et al., 2018), with scores ranging from 0 to 8, with a score of 6 or more being considered high quality, 4–5 being considered moderate quality, and 3 or less being considered low quality. The results of the quality assessment for each study are shown in Supplementary Table S2.

### Data analysis

The extracted data were analyzed via the *R* language package 4.3.2 and combined to calculate the drug resistance rate of *C. acnes*. A *p* value less than 0.05 was considered statistically significant in all analyses. Pooled estimates and 95% confidence intervals (CIs) were calculated via the fixed-effect model of the Mantel–Haenszel method and the random-effect model of the DerSimonian–Laird method (DerSimonian and Laird, 1986; Kuritz et al., 1988). The heterogeneity between studies was evaluated via Cochran’s *Q* test and the inverse variance index (*I*^2^). The *I*^2^ statistic, which is employed to quantify inconsistency, was utilized to assess the degree of variation across studies, thereby reflecting the extent of heterogeneity (von Hippel, 2015). The assumptions that *I*^2^ is less than 25%, between 25 and 75%, and greater than 75% indicate low, moderate, and high degrees of inter-study heterogeneity, respectively (Higgins et al., 2003). Given that antibiotic resistance rates can vary widely across the globe, random effects models are considered more appropriate (Borenstein et al., 2010). The results of fixed effects models are also presented to make the results more comprehensive and robust.

To investigate potential sources of variability, subgroup analyses were conducted. Differences in the prevalence of resistance rates in different regions were analyzed by subgrouping resistance rates across different continents, countries, and different provinces in China. Subgroup analyses were also conducted for various susceptibility testing methods to evaluate discrepancies in drug resistance rates. Changes in resistance rates with year were studied by meta-regression analysis, and for studies across years, the year in which isolation of *C. acnes* strains was first initiated in the article was used.

Sensitivity analyses of the included studies were performed to assess the presence of publication bias via funnel plots (Stuck et al., 1998), and significance was assessed via Egger’s test (Egger et al., 1997). The results of the funnel plots of the studies and the Egger test are shown in Supplementary Figure S1.

## Results

### Summary statistics

**Figure 1:**
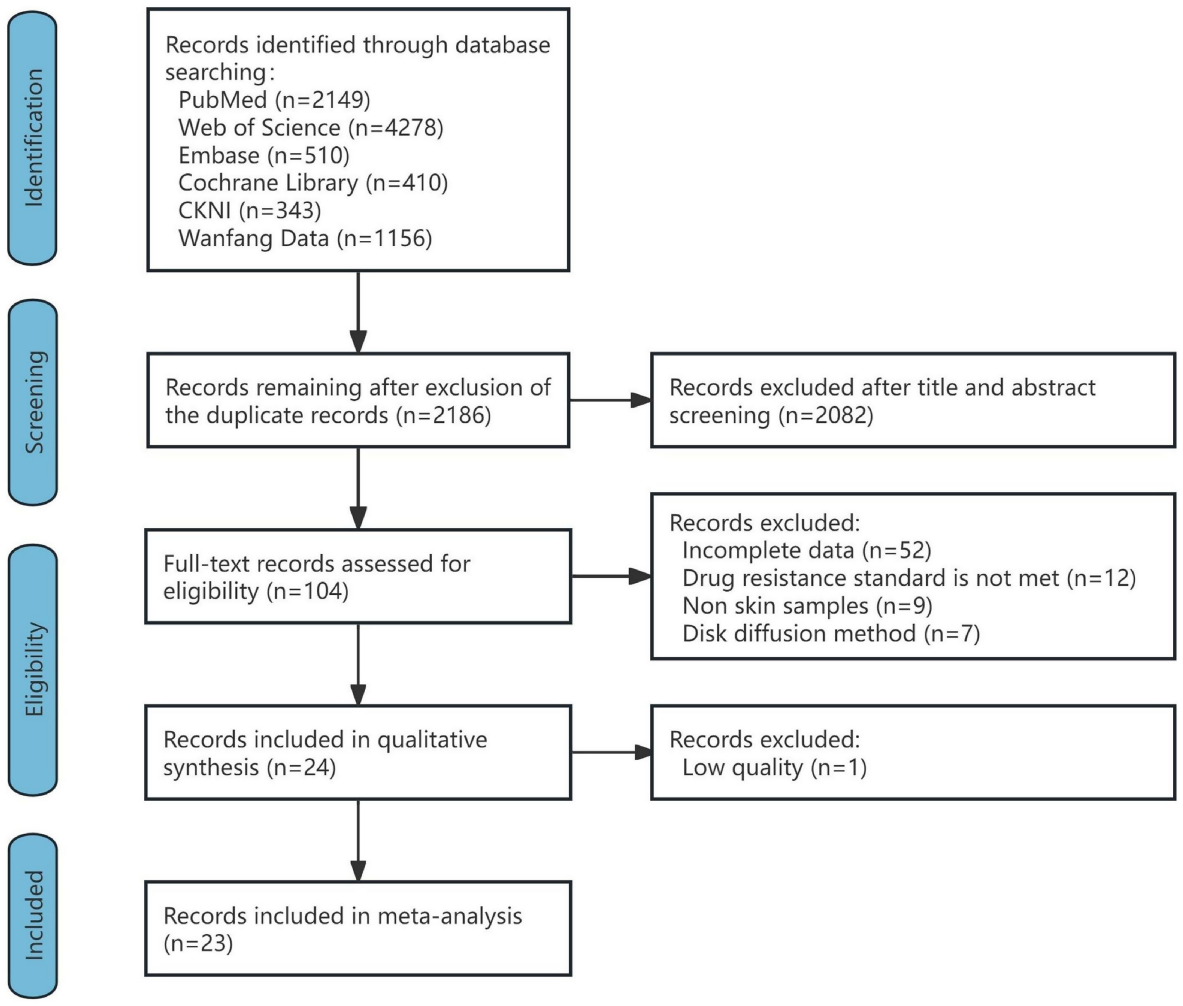
Flow diagram of the study selection process.

A total of 8,846 articles were retrieved. One hundred and four articles were first included by excluding duplicates and reviews, meta-analyses, case reports, commentaries, and reading titles and abstracts of non-*C. acnes* resistance studies, and 80 articles were excluded by reading the full texts of the articles (52 articles with incomplete data, 12 articles with unclear criteria for resistance or nonCLSI or EUCAST criteria, 9 articles with noncutaneous samples, and 7 articles by the disk diffusion method). The remaining 24 articles were evaluated for quality, and 1 article with a quality score of less than 4 was excluded. A total of 23 articles were ultimately included, containing a total of 2,046 *C. acnes* samples. The inter-rater reliability of the included studies between the two authors (C-XZ and B-ZW) was assessed prior to final inclusion. The Cohen Kappa coefficient was 0.83, indicating a high degree of consistency between the studies included by the two authors (C-XZ and B-ZW). The flow chart is shown in Figure 1, and the detailed results of each study are shown in Supplementary Table S3.

### Resistance rates of *Cutibacterium acnes* to various antibiotics

#### Quinolone resistance rates in *Cutibacterium acnes*

**Figure 2:**
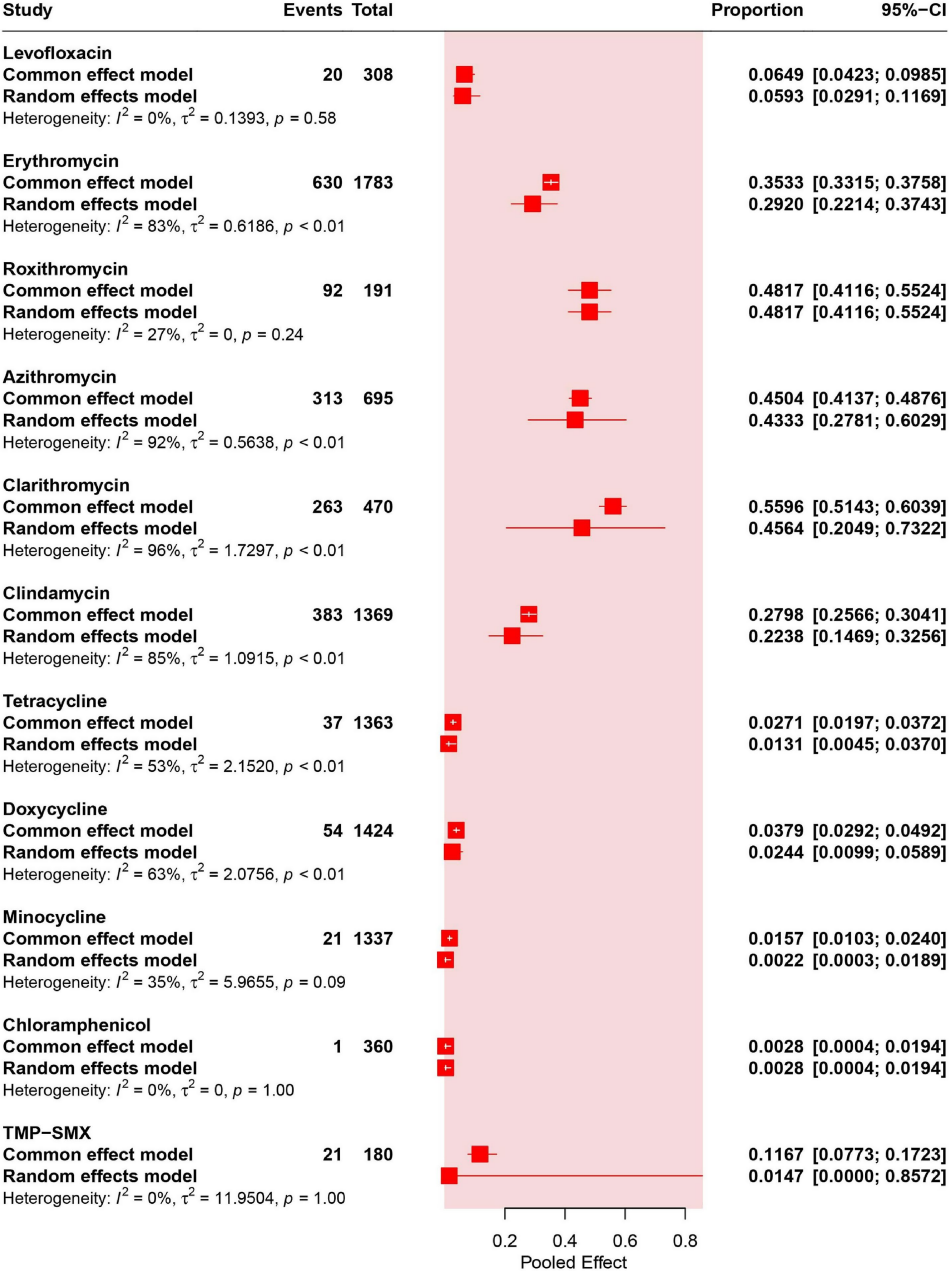
Pooled proportions of antibiotic resistance in *C. acnes.*

**Figure 3:**
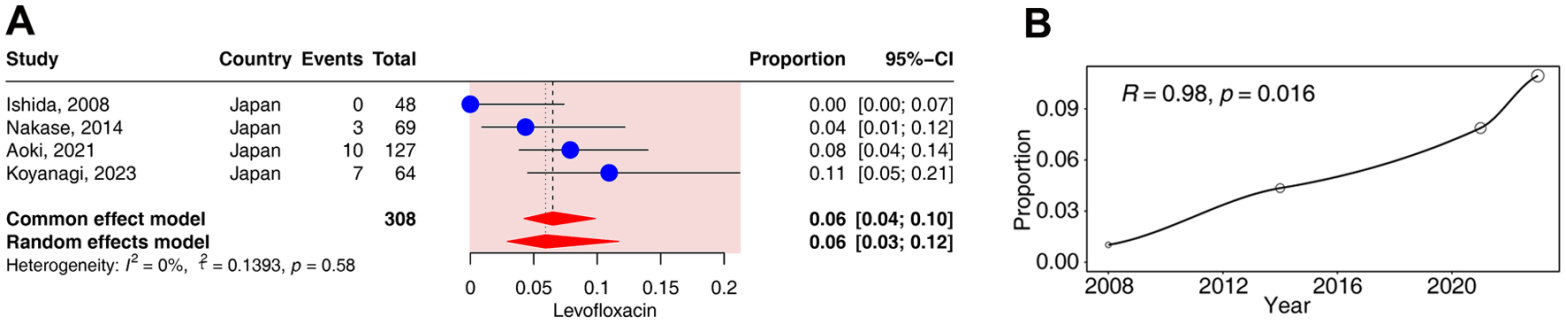
Forest plots and meta-regression analysis plots of levofloxacin resistance rates in *C. acnes*. **(A)** Forest plot of levofloxacin resistance rates in Japan. **(B)** Meta-regression analysis of the temporal trends in the proportion of levofloxacin resistance.

Four studies (corresponding to 308 strains) reported resistance rates to levofloxacin in *C. acnes*. The results have shown a prevalence of levofloxacin- resistance was 5.93% (95% CI: 2.91–11.69%). No heterogeneity was observed in the levofloxacin group (*I*^2^ = 0%) (Figures 2, 3A).

The regression analysis revealed a significant positive correlation between the resistance rates to levofloxacin and time (*R* = 0.98, *p* < 0.05). The resistance rates of levofloxacin gradually increased from 0% in 2008 to 11% in 2023 (Figure 3B).

#### Macrolide resistance rates in *Cutibacterium acnes*

Twenty studies (corresponding to 1783 strains), 2 studies (corresponding to 191 strains), 5 studies (corresponding to 695 strains) and 5 studies (corresponding to 470 strains) reported resistance rates in *C. acnes* to erythromycin, roxithromycin, azithromycin and clarithromycin, respectively. The results have shown a prevalence of erythromycin- resistance was 29.20% (95% CI: 22.14–37.43%), that of roxithromycin- resistance was 48.17% (95% CI: 41.16–55.24%), that of azithromycin- resistance was 43.33% (95% CI: 27.81–60.29%), and that of clarithromycin- resistance was 45.64% (95% CI: 20.49–73.22%). High heterogeneity was observed in the erythromycin, azithromycin and clarithromycin groups (*I*^2^ > 75%), and moderate heterogeneity was observed in the roxithromycin group (*I*^2^ = 27%) (Figure 2).

In the subgroup analyses according to different continents, a significant difference in the resistance rates of azithromycin was observed across continents (*p* < 0.01). The resistance rates of azithromycin ranged from 18% (95% CI: 11–27%) in Europe to 55% (95% CI: 43–67%) in Asia (Supplementary Figure S2A). The resistance rates of erythromycin did not differ significantly across continents (*p* = 0.44) (Supplementary Figure S2B).

**Figure 4:**
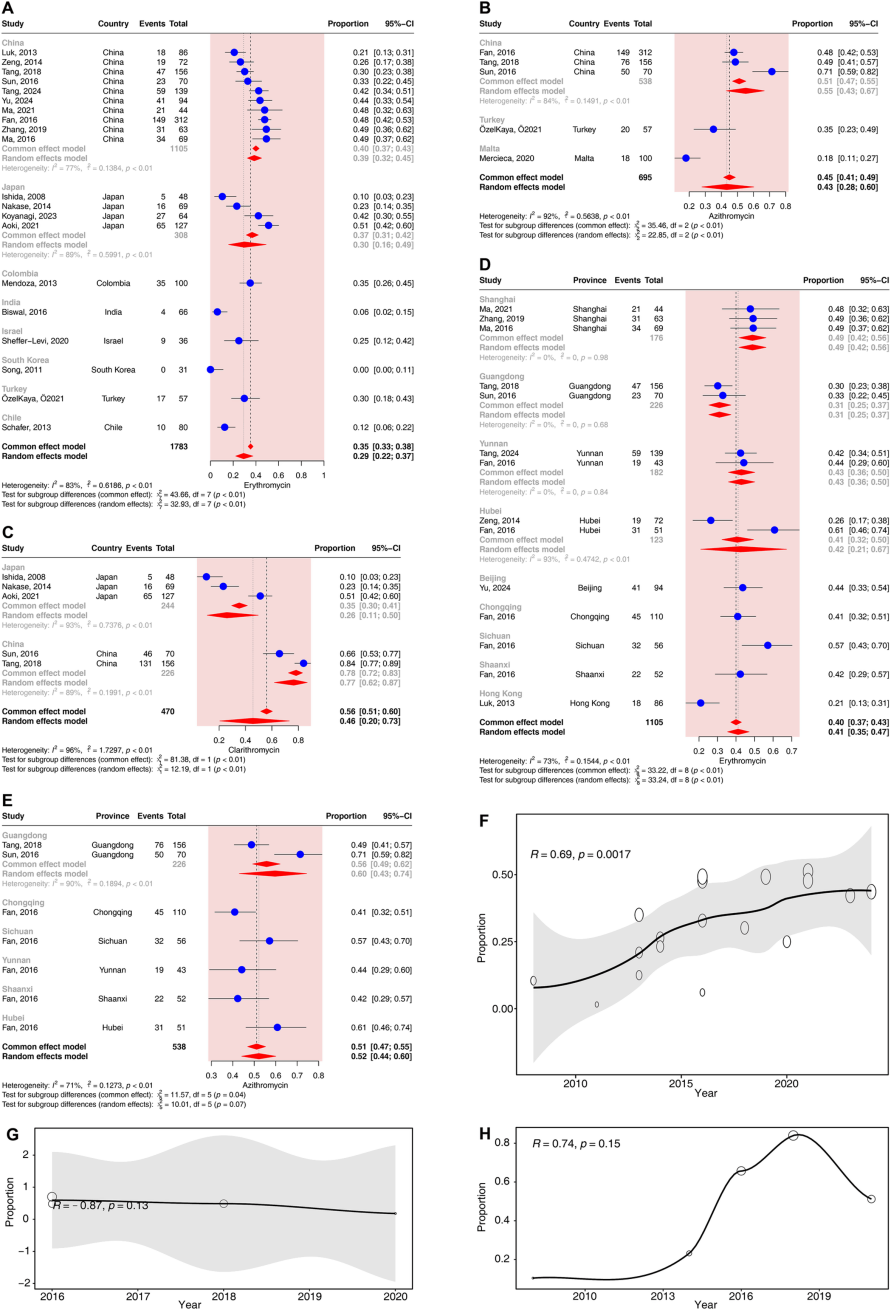
Forest plots and meta-regression analysis plots of macrolide resistance rates in *C. acnes*. **(A)** Forest plot of erythromycin resistance rates in different countries. **(B)** Forest plot of azithromycin resistance rates in different countries. **(C)** Forest plot of clarithromycin resistance rates in different countries. **(D)** Forest plot of erythromycin resistance rates in different provinces of China. **(E)** Forest plot of azithromycin resistance rates in different provinces of China. **(F)** Meta-regression analysis of the temporal trends in the proportion of erythromycin resistance. **(G)** Meta-regression analysis of the temporal trends in the proportion of azithromycin resistance. **(H)** Meta-regression analysis of the temporal trends in the proportion of clarithromycin resistance.

In the subgroup analyses according to different countries, a significant difference in the resistance rates of erythromycin, azithromycin and clarithromycin was observed across countries (*p* < 0.01). The resistance rates of erythromycin ranged from 0% (95% CI: 0–11%) in South Korea to 39% (95% CI: 32–45%) in China (Figure 4A). The resistance rates of azithromycin ranged from 18% (95% CI: 11–27%) in Malta to 55% (95% CI: 43–67%) in China (Figure 4B). The resistance rates of clarithromycin ranged from 26% (95% CI: 11–50%) in Japan to 77% (95% CI: 62–87%) in China (Figure 4C).

In the subgroup analyses according to different provinces in China, a significant difference in the resistance rates to erythromycin was observed across provinces (*p* < 0.01). The resistance rates of erythromycin ranged from 21% (95% CI: 13–31%) in Hong Kong to 57% (95% CI: 43–70%) in Sichuan (Figure 4D). The resistance rates of azithromycin did not differ significantly across provinces (*p* = 0.07) (Figure 4E).

In the subgroup analyses according to different methods of drug susceptibility test, a significant difference in the resistance rates of azithromycin was observed (*p* < 0.01). The resistance rate of azithromycin determined by the agar−dilution method was 51% (95% CI: 39–63%), which was higher than the 18% (95% CI: 11–27%) resistance rate obtained via the *E*-test method (Supplementary Figure S2C). The resistance rates to erythromycin did not differ significantly between the two methods of drug susceptibility test (*p* = 0.17) (Supplementary Figure S2D).

The regression analysis revealed a significant positive correlation between the resistance rates to erythromycin and time (*R* = 0.69, *p* < 0.05). The resistance rates of erythromycin gradually increased from 10% (95% CI: 3–23%) in 2008 to 44% (95% CI: 33–54%) in 2024 (Figure 4F). No significant correlation was detected for the resistance rates of azithromycin and clarithromycin over time (*p* > 0.05) (Figures 4G,H).

#### Lincosamide resistance rates in *Cutibacterium acnes*

Eighteen of the included studies (corresponding to 1,369 strains) reported resistance rates to clindamycin. The results have shown a prevalence of erythromycin resistance was 22.38% (95% CI: 14.69–32.56%). High heterogeneity was observed in the clindamycin group (*I*^2^ = 85%) (Figure 2).

In the subgroup analyses according to different continents, no significant difference in the resistance rates to clindamycin was observed across continents (*p* = 0.08) (Supplementary Figure S3A).

**Figure 5:**
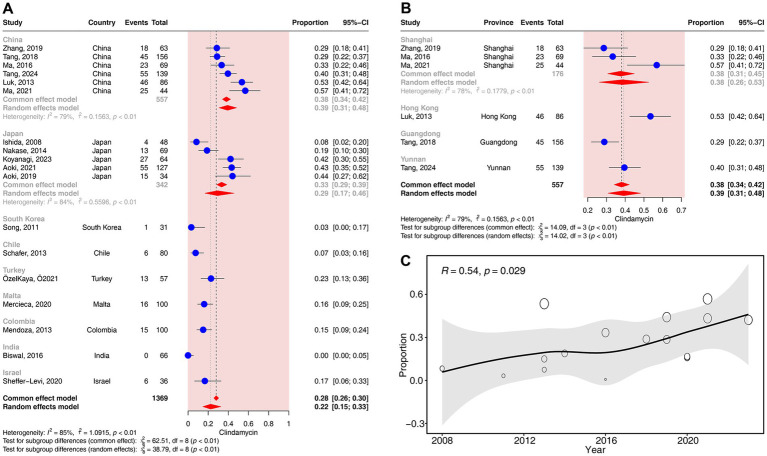
Forest plots and meta-regression analysis plots of lincosamide resistance rates in *C. acnes*. **(A)** Forest plot of clindamycin resistance rates in different countries. **(B)** Forest plot of clindamycin resistance rates in different provinces of China. **(C)** Meta-regression analysis of the temporal trends in the proportion of clindamycin resistance.

In the subgroup analyses according to different countries, a significant difference in the resistance rates to clindamycin was observed across countries (*p* < 0.01). The resistance rates of clindamycin ranged from 0% (95% CI: 0–5%) in India to 39% (95% CI: 31–48%) in China (Figure 5A).

In the subgroup analyses according to different provinces in China, a significant difference in the resistance rates to clindamycin was observed across provinces (*p* < 0.01). The resistance rates of clindamycin ranged from 29% (95% CI: 22–37%) in Guangdong to 53% (95% CI: 42–64%) in Hong Kong (Figure 5B).

In the subgroup analyses according to different methods of drug susceptibility test, the resistance rates to clindamycin did not differ significantly between the two methods of drug susceptibility test (*p* = 0.08) (Supplementary Figure S3B).

The regression analysis revealed a significant positive correlation between the resistance rates to clindamycin and time (*R* = 0.54, *p* < 0.05). The resistance rates of clindamycin gradually increased from 8% (95% CI: 2–20%) in 2008 to 42% (95% CI: 30–55%) in 2023 (Figure 5C).

#### Tetracycline resistance rates in *Cutibacterium acnes*

Fifteen studies (corresponding to 1,363 strains), 14 studies (corresponding to 1,424 strains), and 14 studies (corresponding to 1,337 strains) reported resistance rates to tetracycline, doxycycline, and minocycline, respectively. The results have shown a prevalence of tetracycline resistance was 1.31% (95% CI: 0.45–3.70%), that of doxycycline resistance was 2.44% (95% CI: 0.99–5.89%), and that of minocycline resistance was 0.22% (95% CI: 0.03–1.89%). Moderate heterogeneity was observed in the tetracycline, doxycycline, and minocycline groups (25% < *I*^2^ < 75%) (Figure 2).

In the subgroup analyses according to different continents, no significant difference in the resistance rates to tetracyclines was observed across continents (*p* > 0.05) (Supplementary Figures S4A–C).

**Figure 6:**
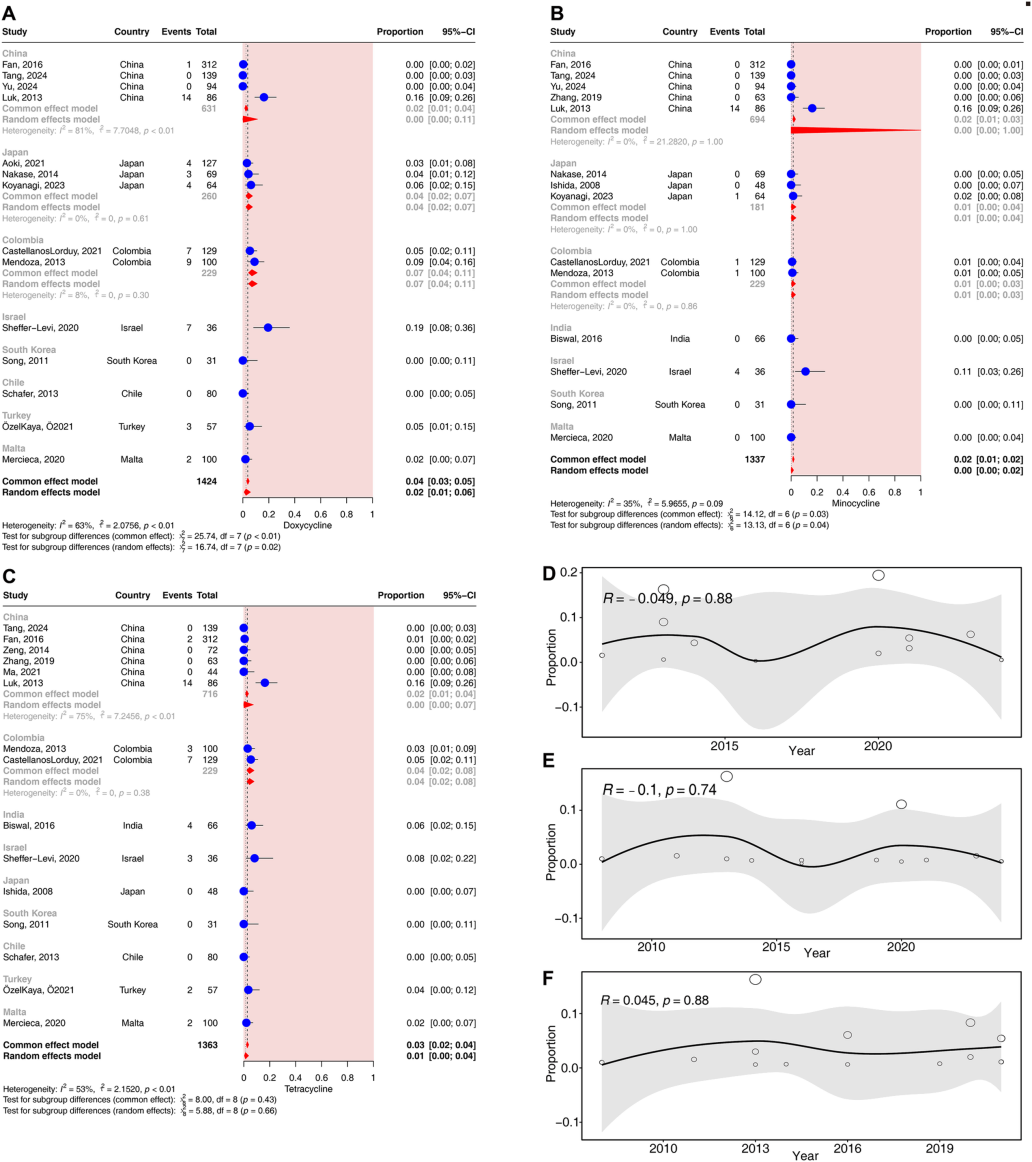
Forest plots and meta-regression analysis plots of tetracycline resistance rates in *C. acnes*. **(A)** Forest plot of doxycycline resistance rates in different countries. **(B)** Forest plot of minocycline resistance rates in different countries. **(C)** Forest plot of tetracycline resistance rates in different countries. **(D)** Meta-regression analysis of the temporal trends in the proportion of doxycycline resistance. **(E)** Meta-regression analysis of the temporal trends in the proportion of minocycline resistance. **(F)** Meta-regression analysis of the temporal trends in the proportion of tetracycline resistance.

In the subgroup analysis according to different countries, significant differences in the rates of resistance to doxycycline and minocycline were observed across countries (*p* < 0.05). The resistance rates of doxycycline ranged from 0% (95% CI, 0–5%) in Chile to 19% (95% CI, 8–36%) in Israel (Figure 6A). The resistance rates to minocycline ranged from 0% (95% CI, 0–4%) in Malta to 11% (95% CI: 3–26%) in Israel (Figure 6B). No significant difference in the resistance rates to tetracycline was observed across countries (*p* = 0.66) (Figure 6C).

In the subgroup analyses according to different methods of drug susceptibility test, the resistance rates to tetracyclines did not differ significantly between the two methods of drug susceptibility test (*p* > 0.05) (Supplementary Figures S4D–F).

No significant correlation was detected for the resistance rates to tetracyclines over time (*p* > 0.05) (Figures 6D–F).

#### Chloramphenicol resistance rates in *Cutibacterium acnes*

Two studies (corresponding to 360 strains) reported resistance rates to chloramphenicol. The results have shown a prevalence of chloramphenicol resistance was 0.28% (95% CI: 0.04–1.94%). No heterogeneity was observed in the chloramphenicol group (*I*^2^ = 0%) (Figure 2).

In the subgroup analyses according to different countries, no significant difference in the resistance rates to chloramphenicol was observed across countries (*p* > 0.05) (Supplementary Figure S5A).

In the subgroup analyses according to different provinces in China, no significant difference in the resistance rates to chloramphenicol was observed across provinces (*p* > 0.05) (Supplementary Figure S5B).

#### TMP-SMX resistance rates in *Cutibacterium acnes*

Two studies (corresponding to 180 strains) reported the resistance rates of TMP-SMX. The results have shown a prevalence of TMP-SMX resistance was 1.47% (95% CI: 0.00–85.72%) (Supplementary Figure S6A). No heterogeneity was observed in the TMP-SMX group (*I*^2^ = 0%) (Figure 2).

In the subgroup analyses according to different countries, no significant difference in the resistance rates of TMP-SMX was observed across countries (*p* > 0.05) (Supplementary Figure S6A).

In the subgroup analyses according to different methods of drug susceptibility test, no statistically significant differences were observed in the rates of resistance to TMP-SMX (*p* > 0.05) (Supplementary Figure S6B).

#### Publication bias

The presence of publication bias was assessed via funnel plots and tested for significance via Egger’s test, with a *p* value < 0.05 considered significant, and Egger’s test was applicable only to studies containing more than 10 items. The *p* values for the erythromycin, clindamycin and tetracyclines egger’s tests were less than 0.05, which were significant. Funnel plots and Egger’s test results are shown in the Supplementary Figure S1.

## Discussion

Acne is a chronic inflammatory disease that affects mainly adolescents and is most often characterized by pimples, papules, pustules, and nodules. Acne is a multifactorial disease caused by the interaction of environmental and genetic factors (Dall'Oglio et al., 2020), and its pathogenesis is closely related to the colonization of *C. acnes*, which is considered the most likely causative pathogen, as it was first observed in acne lesions by Unna (1896) and was subsequently isolated by Lee et al. (2019). Previous studies have shown that *C. acnes* can produce inflammatory factors such as IL-1, IL-6, and IL-8 through the activation of toll-like receptors on keratinocytes, which in turn activate the MAPK and NF-kB pathways, subsequently inducing inflammation (Nagy et al., 2005). In addition, the recognition of *C. acnes* by TLR-2 receptors on sebocytes can also activate the NF-kB pathway, leading to inflammation (Cong et al., 2019). A range of immune responses associated with *C. acnes* also involve CD4 + T lymphocytes, especially Th1 and Th17 cells (Agak et al., 2014).

Currently, antibiotic therapy may be used as a first-line treatment to manage moderate-to-severe acne (Thiboutot et al., 2020); however, owing to the widespread use of antibiotics in the clinical practice in human medicine, especially macrolides and tetracyclines, a year-to-year increase in resistance to *C. acnes* has been reported in some countries and regions (Yang et al., 2018; Zhu et al., 2019)^,^ and with the emergence of cross-resistance, timely assessment of the prevalence of *C. acnes* drug resistance and the emergence of cross-resistance is urgently needed.

In this study, *C. acnes* presented a greater prevalence of resistance to macrolides in comparison to other antibiotics. The resistance mechanisms responsible for macrolide resistance in bacterial pathogens may involve genetic mutations and modifications of the 23S rRNA, macrolide efflux systems, as well as inactivation of macrolides by phosphotransferases and esterases (Ogawara, 2019). In addition, mutations in the 23S rRNA gene may also contribute to clindamycin resistance (Kulczycka-Mierzejewska et al., 2018). *In vitro* induction experiments revealed a potential association between 23S rRNA mutations and antibiotic exposure. Consequently, prolonged use of a single antibiotic may be a contributing factor to the prevalence of resistant strains (Nakase et al., 2018). The resistance rates to erythromycin, clarithromycin, and azithromycin in China were notably higher than those reported in other countries, which is in line with the results previously reported by Zhu et al. (2019). In addition, the resistance rate of erythromycin in Sichuan Province was significantly higher than that in other regions of China. Acne treatment in China has long relied on macrolides and clindamycin as topical antibiotics, especially for mild to moderate acne, local antibiotic monotherapy is overused, which may lead to an increase in the resistance rate of macrolides (Ma et al., 2016). In addition, regression analyses of erythromycin revealed a positive correlation between resistance rates and the elapse of time (in years), which aligns with prior research findings (Beig et al., 2024). Consequently, it is crucial to closely monitor macrolide resistance rates and avoid the prolonged use of a single antibiotic in acne treatment.

Resistance to lincomycin-based drugs in *C. acnes* is of similar concern, with studies including only studies on clindamycin. The rate of resistance to clindamycin was also high in comparison to antibiotics other than macrolides, which is consistent with the results of a previous Jordanian study of 100 individuals (Alkhawaja et al., 2020). This study revealed that the resistance rate to clindamycin was significantly higher in China than in other regions, especially Hong Kong. In addition, the regression analysis revealed a statistically significant correlation between the increase in the clindamycin resistance rate and time. A prior study conducted in Japan reported a similar upwards trend in clindamycin resistance rates (Nakase et al., 2017a,b). Previous studies have shown that the *erm*(X) gene may play a role in antibiotic resistance in *C. acnes*. The enzymatic methylation of adenosine in the 23S rRNA ribosomal subunit is encoded by genes belonging to the *erm* family, which confer antibiotic resistance (Szemraj et al., 2018). In addition, the *erm*(X) gene, located on the transposon Tn5432, is considered to be transmitted among *C. acnes* strains by conjugation (Aoki et al., 2019). Another study also reported the *erm*(X) gene on a transferable line plasmid (pTP-CU411). This mechanism may play a significant role in the emergence and dissemination of clindamycin-resistant *C. acnes* among acne patients (Koizumi et al., 2022).

The resistance rate of *C. acnes* to quinolones was relatively low, and the *C. acnes* isolates included in this study were all from Japan. Consistently low resistance rates were reported in a previous trial of quinolone resistance in *C. acnes* (Nenoff et al., 2004). The Japanese acne treatment guidelines recommend doxycycline and minocycline as first-line agents, with roxithromycin and faropenem as subsequent options, and levofloxacin as a later-stage choice (Hayashi et al., 2018). Despite the low rate of levofloxacin resistance, regression analysis revealed a positive correlation between the rate of levofloxacin resistance and the yearly increase. *In vitro* experiments have demonstrated that *C. acnes* can develop resistance via progressive exposure to levofloxacin (Nakase et al., 2016). Therefore, the long-term use of levofloxacin may be the cause of the increased resistance rate in Japan.

The U. S. guidelines for the treatment of common acne state that tetracyclines (especially doxycycline and minocycline) are often recommended as first-line therapeutic agents for acne (Reynolds et al., 2024), which, in addition to their own antibacterial effects, may also have anti-inflammatory effects through the inhibition of neutrophil chemotaxis and the reduction of inflammatory cytokines (Perret and Tait, 2014). This study revealed that the resistance rates of *C. acnes* to tetracyclines were relatively low, with minocycline exhibiting a particularly low resistance rate. These findings are consistent with the results reported in a previous study conducted in Colombia (Castellanos Lorduy et al., 2021). Regression analysis also did not reveal a correlation between tetracyclines resistance and the yearly increase in prevalence. However, in one of the included studies, there was an increase in the prevalence of doxycycline-resistant strains, with yearly increases in prevalence (Aoki et al., 2021). The resistance mechanism of *C. acnes* to tetracyclines may be attributed to the following factors. Previous studies have shown that 16S rRNA is an important component of the 30S subunit of bacterial ribosomes and that tetracycline antibiotics may inhibit the initiation phase of protein synthesis by binding to a specific region of 16S rRNA (Barrenechea et al., 2021). Some strains of *C. acnes* were found to have 16S rRNA mutations. When the 16S rRNA gene is mutated, it may interfere with the ability of tetracycline to bind to the ribosome, thereby reducing drug susceptibility (Aoki et al., 2021). In addition, amino acid substitutions in the S10 protein may enable *C. acnes* to develop resistance to tetracyclines. The S10 protein is encoded by the *rpsJ* gene and is a structural protein of the 30S subunit of the ribosome. Amino acid substitutions in the S10 protein may compromise the ability of tetracyclines to bind to 16S rRNA, thereby reducing drug sensitivity (Sutcliffe et al., 2020). Furthermore, the amino acid substitution in the S10 protein may precede the mutation in the 16S rRNA. Certain strains acquire additional 16S rRNA mutations following S10 mutation, resulting in a significant increase in the MIC (Nakase et al., 2017a,b). *Tet*(W) belongs to the ribosome protective protein (RPP) gene family, which encodes Tet(W) proteins that may mediate drug resistance by binding to ribosomes and unblocking tetracycline (Connell et al., 2003). A prior study revealed that some *C. acnes* strains harbor the foreign resistance gene *tet*(W), and these strains exhibit varying levels of sensitivity to doxycycline depending on the expression level of *tet*(W) (Aoki et al., 2021). Since tetracycline antibiotics serve as first-line treatments for acne in many countries, closely monitoring trends in tetracycline resistance rates is crucial.

Notably, first, chloramphenicol was included in relatively few studies, and there may be bias, but only one strain of *C. acnes* was resistant to chloramphenicol in the included studies (360 strains in total), which suggests that *C. acnes* may be less resistant to chloramphenicol at the present time. Second, although the resistance rate of *C. acnes* to TMP-SMX was relatively low in this study, prior research has indicated that *C. acnes* is highly resistant to sulfonamides (Fan et al., 2016; Nagy et al., 2018). Given the limited number of studies included in the TMP-SMX group, this result needs to be viewed with great caution.

This study also compared the differences in drug resistance rates measured via the agar-dilution method and the *E*-test method. The results indicated that a significant difference in the drug resistance rate was observed only for azithromycin, whereas no significant differences were noted for the other antibiotics. Both of these methods are applicable for evaluating the susceptibility of anaerobic bacteria (Gajdacs et al., 2017).

Cross-resistance to different antibiotics was found in some of the included and excluded studies, with most studies finding cross-resistance to ML (Ishida et al., 2008; Abdel Fattah and Darwish, 2013; Luk et al., 2013; Mendoza et al., 2013; Schafer et al., 2013; Fan et al., 2016; Ma et al., 2016; Nakase et al., 2020). The mechanisms of resistance to ML in *C. acnes* are likely mediated by 23S rRNA mutations and methylation of 23S rRNA via the ribosomal methylase gene *erm*(X) (Ross et al., 2002; Ross et al., 2003). This is consistent with the high rate of resistance found for both macrolides and clindamycin in the results of this study. The presence of cross-resistance was also found for tetracyclines (Abdel Fattah and Darwish, 2013; Luk et al., 2013; Mendoza et al., 2013; Sheffer-Levi et al., 2020). In addition, some studies have shown that cross-resistance also occurs between ML and tetracyclines (Abdel Fattah and Darwish, 2013; Luk et al., 2013; Sheffer-Levi et al., 2020). Aoki et al. identified the presence of a plasmid DNA designated pTZC1, which carries the novel ML resistance gene *erm*(50) and the tetracycline resistance gene *tet*(W). This plasmid, pTZC1, is likely capable of horizontal transfer among *C. acnes* strains, conferring resistance to ML and tetracyclines (Aoki et al., 2020). In addition to the gene mutations mentioned above and the acquisition of new genes, the activation of efflux pumps can concurrently expel a variety of antibiotics with diverse structures. A previous study has demonstrated that the overexpression of efflux pumps in tetracycline-resistant strains not only decreases the intracellular concentration of tetracycline compounds but also may impact the efficacy of other antibiotics that depend on proton gradients for cellular entry (Nakase et al., 2017a,b; Zhang et al., 2017). In addition, biofilms formed by drug-resistant strains may enhance bacterial adaptive resistance through mechanisms such as restricting antibiotic penetration, inducing the formation of dormant bacterial populations (Jahns et al., 2016). The inappropriate and excessive use of antibiotics can contribute to the emergence and spread of multidrug-resistant bacteria (Di Lodovico et al., 2022).

The included studies also examined the effect of treatment history on the MIC/resistance rate of *C. acnes*, and some of the studies reported a correlation between antimicrobial use and the MIC/resistance rate (Dumont-Wallon et al., 2010; Song et al., 2011; Schafer et al., 2013; Fan et al., 2016; Karadag et al., 2021), as evidenced by the findings that there was a correlation between a history of tetracycline use and the MIC/resistance rate (Dumont-Wallon et al., 2010; Song et al., 2011; Fan et al., 2016; Nakase et al., 2020) and that there was a correlation between a history of ML use and the MIC/resistance rates (Dumont-Wallon et al., 2010; Schafer et al., 2013; Nakase et al., 2020)^.^ The results of this study revealed that Asia presented higher rates of antibiotic resistance in the ML class than did the other continents. China presented higher rates of antibiotic resistance in the ML class than did the other countries did, and a previous study investigated the correlation between rates of antibiotic resistance and reported sales of antimicrobial medicines in the eight European countries for which these data were reported. In European Union countries, MLS antibiotic resistance rates are correlated with the sales of their drugs (Oprica and Nord, 2005); thus, the abovementioned regions with higher resistance rates may be due to the misuse of such antibiotics in the region, but some studies have reported no correlation between antibiotic use history and MIC/resistance rates (Sheffer-Levi et al., 2020; Koyanagi et al., 2023). In addition, a study reported significant differences in the percentage of resistance to oral versus topical antibiotics, with higher rates of resistance observed for topical antibiotics (Toyne et al., 2012).

Our study has several limitations. First, the majority of included studies originated from Asian regions, particularly China, which may introduce publication bias. Second, upon meta-analysis of pooled data, substantial heterogeneity was observed that could not be fully explained by regional differences or variations in drug susceptibility testing methods. This heterogeneity may be influenced by patient-specific factors such as age, sex, medication history, and disease duration; however, insufficient data were available to assess these contributions definitively. Third, certain regions were underrepresented in the study, potentially exacerbating publication bias and introducing regional heterogeneity that was not fully captured. Future studies should prioritize larger, more geographically diverse datasets to address these limitations.

The novelties of this study are as follows: we minimized the heterogeneity of the studies through more rational inclusion and exclusion criteria, including limited resistance criteria and methods of drug susceptibility test, conducted a more detailed regional breakdown of the subgroup analyses, performed regression analyses of the resistance rate of each antibiotic with respect to the year, and further generalized the relationship between cross-resistance and history of antibiotic use and the antibiotic resistance rate.

In summary, this research revealed differences in the resistance rates of different antibiotics, the diversity of the prevalence of antibiotic resistance in different regions, and the increase in some antibiotic resistance rates annually. This study highlights the complex prevalence of antibiotic resistance, which reflects the necessity of continuous monitoring of antibiotic resistance rates. The present study improves the systematic review and meta-analysis of resistance rates of *C. acnes* and provides a basis for the further rational use of antibiotics for the treatment of acne.

## Conclusion

This study aimed to systematically assess the antibiotic resistance rates of *C. acnes* isolates from patients with acne through a meta-analysis approach, thereby providing a rational basis for optimizing antibiotic treatment strategies in acne management. Key findings revealed significantly elevated resistance rates of *C. acnes* to macrolides and clindamycin, particularly in China, which were notably higher compared to other regions. This disparity is likely attributable to the overuse of these antibiotics in clinical practice. Conversely, resistance rates to tetracyclines remain relatively low, supporting their continued use as first-line therapeutic options. Furthermore, temporal analysis revealed increasing resistance rates to levofloxacin, erythromycin, and clindamycin, underscoring the critical importance of prudent antibiotic stewardship. The regional variation in resistance rates highlights the necessity of implementing region-specific surveillance systems and tailoring antibiotic guidelines accordingly. These findings emphasize the urgent need to refine current antibiotic usage strategies and explore alternative treatment modalities to mitigate the spread of antimicrobial resistance. Future study should prioritize the analysis of larger, more geographically diverse datasets to elucidate the underlying factors contributing to variations in resistance rates. In summary, this study underscores the complex and regionally heterogeneous nature of *C. acnes* antibiotic resistance, advocating for continuous monitoring and evidence-based antibiotic prescribing practices to ensure effective acne management while minimizing resistance risk.

## Data Availability

The data that support the findings of this study are available from the corresponding author upon reasonable request.
