# Supplementary material for: Antibiotic resistance rates in Cutibacterium acnes isolated from patients with acne vulgaris: a systematic review and meta-analysis
Source: Front Microbiol. 2025 Jun 4;16:1565111. doi: 10.3389/fmicb.2025.1565111 (PMC12174412; doi:10.3389/fmicb.2025.1565111)
Supplement: Supplementary file 1 [file Data_Sheet_1.docx]

**Supplementary Materials**

**Table S1 Retrieve the keywords used**

(Acne Vulgaris OR Acne OR Acnes OR Cutibacterium OR Cutibacterium acnes OR C. acnes OR Propionibacterium OR Propionibacterium acnes OR P. acnes) AND (Drug Resistance, Microbial OR Drug Resistances, Microbial OR Antimicrobial Drug Resistance OR Antimicrobial Drug Resistances OR Antibiotic Resistance, Microbial OR Antibiotic Resistance OR Resistance, Antibiotic OR Drug Resistance, Bacterial OR Antibacterial Drug Resistance OR Antibiotic Resistance, Bacterial OR Anti-Bacterial Agents OR Agents, Anti-Bacterial OR Anti Bacterial Agents OR Antibacterial Agents OR Agents, Antibacterial OR Antibacterial Agent OR Agent, Antibacterial OR Anti-Bacterial Compounds OR Anti Bacterial Compounds OR Compounds, Anti-Bacterial OR Anti-Bacterial Agent OR Agent, Anti-Bacterial OR Anti Bacterial Agent OR Anti-Bacterial Compound OR Anti Bacterial Compound OR Compound, Anti-Bacterial OR Antibiotics OR Antibiotic OR Microbial Sensitivity Tests OR Microbial Sensitivity Test OR Sensitivity Test, Microbial OR Sensitivity Tests, Microbial OR Test, Microbial Sensitivity OR Tests, Microbial Sensitivity OR Drug Sensitivity Assay, Microbial OR Antimicrobial Susceptibility Breakpoint Determination OR Breakpoint Determination, Antimicrobial Susceptibility OR Bacterial Sensitivity Tests OR Tests, Bacterial Sensitivity OR Sensitivity Tests, Bacterial OR Test, Bacterial Sensitivity OR Bacterial Sensitivity Test OR Sensitivity Test, Bacterial)

| Part | MeSH | Entry Terms |
| --- | --- | --- |
| Part 1 | Acne Vulgaris | Acne  Acnes |
|  | Cutibacterium |  |
|  | Cutibacterium acnes | C. acnes |
|  | Propionibacterium |  |
|  | Propionibacterium acnes | P. acnes |
| Part 2 | Drug Resistance, Microbial | Drug Resistances, Microbial  Antimicrobial Drug Resistance  Antimicrobial Drug Resistances  Antibiotic Resistance, Microbial  Antibiotic Resistance  Resistance, Antibiotic |
|  | Drug Resistance, Bacterial | Antibacterial Drug Resistance  Antibiotic Resistance, Bacterial |
|  | Anti-Bacterial Agents | Agents, Anti-Bacterial  Anti Bacterial Agents  Antibacterial Agents  Agents, Antibacterial  Antibacterial Agent  Agent, Antibacterial  Anti-Bacterial Compounds  Anti Bacterial Compounds  Compounds, Anti-Bacterial  Anti-Bacterial Agent  Agent, Anti-Bacterial  Anti Bacterial Agent  Anti-Bacterial Compound  Anti Bacterial Compound  Compound, Anti-Bacterial  Antibiotics  Antibiotic |
|  | Microbial Sensitivity Tests | Microbial Sensitivity Test  Sensitivity Test, Microbial  Sensitivity Tests, Microbial  Test, Microbial Sensitivity  Tests, Microbial Sensitivity  Drug Sensitivity Assay, Microbial  Antimicrobial Susceptibility Breakpoint Determination  Breakpoint Determination, Antimicrobial Susceptibility  Bacterial Sensitivity Tests  Tests, Bacterial Sensitivity  Sensitivity Tests, Bacterial  Test, Bacterial Sensitivity  Bacterial Sensitivity Test  Sensitivity Test, Bacterial |

**Table S2 Results of the quality evaluation of each study**

| Studies | 1. Was the study’s target population a close  representation of  the target  population? | 2. Were the study participants  recruited in an  appropriate way? | 3. Was the sample size adequate? | 4. Were the study  subjects’  characteristics described in  detail? | 5. Was the data analysis  conducted with sufficient  coverage of the identified  sample? | 6. Were objective, standard criteria  used for the  measurement of the condition? | 7. Was the condition measured reliably? | 8. Are all important confounding  factors/subgroups/differences identified and accounted for? | Total score |
| --- | --- | --- | --- | --- | --- | --- | --- | --- | --- |
| Fan2016 [1] | Y | Y | Y | Y | Y | Y | Y | N | 7 |
| Mendoza2013 [2] | Y | Y | N | Y | Y | Y | Y | Y | 7 |
| Biswal2016 [3] | N | Y | N | Y | Y | Y | Y | N | 5 |
| Sheffer-Levi2020 [4] | N | Y | N | Y | Y | Y | Y | Y | 6 |
| Nakase2014 [5] | N | Y | N | Y | Y | Y | Y | Y | 6 |
| Koyanagi2023 [6] | Y | Y | N | N | Y | Y | Y | Y | 6 |
| Aoki2021 [7] | N | Y | N | Y | Y | Y | Y | Y | 6 |
| Ishida2008 [8] | N | Y | N | N | Y | Y | Y | N | 4 |
| Song2011 [9] | N | Y | N | N | Y | Y | Y | Y | 5 |
| Luk2013 [10] | N | Y | N | Y | Y | Y | Y | Y | 6 |
| Schafer2013 [11] | N | Y | N | Y | Y | Y | Y | Y | 6 |
| Ma2016 [12] | N | Y | N | Y | Y | Y | Y | Y | 6 |
| Tang2018 [13] | N | Y | N | Y | Y | Y | Y | N | 5 |
| Ma2021 [14] | N | Y | N | N | Y | Y | Y | N | 4 |
| Sun2016 [15] | N | Y | N | N | Y | Y | Y | N | 4 |
| Tang2024 [16] | N | Y | N | Y | Y | Y | Y | Y | 6 |
| Zeng2014 [17] | N | Y | N | Y | Y | Y | Y | Y | 6 |
| Özel Kaya2021[18] | N | Y | N | Y | Y | Y | Y | N | 5 |
| Aoki2019[19] | N | Y | N | Y | Y | Y | Y | N | 5 |
| Mercieca2020[20] | Y | Y | N | N | Y | Y | Y | Y | 6 |
| Castellanos Lorduy2021[21] | N | Y | N | Y | Y | Y | Y | Y | 6 |
| Yu2024[22] | Y | Y | N | N | Y | Y | Y | Y | 6 |
| Zhang2019[23] | Y | Y | N | N | Y | Y | Y | Y | 6 |

N, no; NA, not applicable; Y, yes

**Quality Assessment of Included Studies**

1. Was the study’s target population a close representation of the target population? Y/N

The target population refers to the group people entities to which the results of the study will be generalized. Studies providing data from several hospitals across the nation can be well representative of the national situation in terms of resistance prevalence.

2. Were the study participants recruited in an appropriate way? Y/N Prospective consecutive enrollment (no random selection)

3. Was the sample size adequate? Y/N

We considered adequate studies computing their own sample size calculation or, if not mentioned, we deemed as adequate studies including at least 250 isolates.

4. Were the study subjects’characteristics described in detail? Y/N

The question aims at assessing whether and how the sociodemographic features (age, sex, and race) and clinical features were reported in the study.

5. Was the data analysis conducted with sufficient coverage of the identified sample? Y/N Are resistance results provided for all the isolates?

6. Were objective, standard criteria used for the measurement of the condition? Y/N

Several diagnostics tools were used with satisfying sensitivity and specificity % in reporting prevalence data. New and not well-defined diagnostic technique can provide unreliable data.

7. Was the condition measured reliably? Y/N

The type of antimicrobial susceptibility testing (phenotypic and/or genotypic) and the breakpoint systems referred to the definition of resistance should be reported.

8. Are all important confounding factors/subgroups/differences identified and accounted for? Y/N

The type of resistance (primary or secondary) should be reported. Previous administration of antibiotics/anti-acid should be reported. In case of secondary resistance, the previous antibiotic treatment should be provided.

The following points were deemed as redundant for the purpose of this systematic review.

1. Was there appropriate statistical analysis? NA

2. Were subpopulations identified using objective criteria? NA N, no; NA, not applicable; Y, yes

N, no; NA, not applicable; Y, yes

**Studies Included in the Systematic Review and Meta-Analysis**

[1]. Fan, Y., et al., Multicenter cross-sectional observational study of antibiotic resistance and the genotypes of Propionibacterium acnes isolated from Chinese patients with acne vulgaris. J Dermatol, 2016. 43(4): p. 406-13.

[2]. Mendoza, N., et al., Antimicrobial susceptibility of Propionibacterium acnes isolates from acne patients in Colombia. Int J Dermatol, 2013. 52(6): p. 688-92.

[3]. Biswal, I., et al., In vitro antimicrobial susceptibility patterns of Propionibacterium acnes isolated from patients with acne vulgaris. J Infect Dev Ctries, 2016. 10(10): p. 1140-1145.

[4]. Sheffer-Levi, S., et al., Antibiotic Susceptibility of Cutibacterium acnes Strains Isolated from Israeli Acne Patients. Acta Derm Venereol, 2020. 100(17): p. adv00295.

[5]. Nakase, K., et al., Relationship between the severity of acne vulgaris and antimicrobial resistance of bacteria isolated from acne lesions in a hospital in Japan. J Med Microbiol, 2014. 63(Pt 5): p. 721-728.

[6]. Koyanagi, S., et al., Increased frequency of clindamycin-resistant Cutibacterium acnes strains isolated from Japanese patients with acne vulgaris caused by the prevalence of exogenous resistance genes. J Dermatol, 2023. 50(6): p. 793-799.

[7]. Aoki, S., et al., Increased prevalence of doxycycline low-susceptible Cutibacterium acnes isolated from acne patients in Japan caused by antimicrobial use and diversification of tetracycline resistance factors. J Dermatol, 2021. 48(9): p. 1365-1371.

[8]. Ishida, N., et al., Antimicrobial susceptibilities of Propionibacterium acnes isolated from patients with acne vulgaris. Microbiol Immunol, 2008. 52(12): p. 621-4.

[9]. Song, M., et al., Antibiotic susceptibility of Propionibacterium acnes isolated from acne vulgaris in Korea. J Dermatol, 2011. 38(7): p. 667-73.

[10]. Luk, N.T., et al., Antibiotic-resistant Propionibacterium acnes among acne patients in a regional skin centre in Hong Kong. J Eur Acad Dermatol Venereol, 2013. 27(1): p. 31-6.

[11]. Schafer, F., et al., Antimicrobial susceptibility and genetic characteristics of Propionibacterium acnes isolated from patients with acne. Int J Dermatol, 2013. 52(4): p. 418-25.

[12]. Ma, Y., et al., Antimicrobial activity of topical agents against Propionibacterium acnes: an in vitro study of clinical isolates from a hospital in Shanghai, China. Front Med, 2016. 10(4): p. 517-521.

[13]. Tang Huijing et al., Phenotype analysis of clinical resistance of Propionibacterium acnes to macrolide-lincoamide antibiotics. Journal of Dermatology and Venereology, 2018. 25(05):p. 261-264.

[14]. Ma Ying et al., Correlation between biofilm formation and antimicrobial resistance of Propionibacterium acnes. Chinese Journal of Infection and Chemotherapy, 2021. 21(06):p. 703-707.

[15]. Sun Fei et al., Sensitivity analysis of propionibacterium acnes to macrolide antibiotics in acne patients. Journal of Dermatology and Venereology, 2016. 23(04): p. 256-258.

[16]. Tang Wanjuan et al., Analysis of resistance to common antibiotics of acne patients by isolated bacteria from skin lesions. Chinese Journal of Dermatology and Venereology, 2024. 38(07):p. 740-747.

[17]. Zeng Xianyu et al., Drug resistance analysis of propionibacterium isolated from skin lesions of acne patients in Wuhan. Chinese Journal of Dermatology and Venereology, 2014. 28(02): p.131-134

[18]. Ozel Kaya, N., et al., [Phylotyping and Determining the Antimicrobial Susceptibility of Cutibacterium acnes Isolated from Patients with Acne Vulgaris]. Mikrobiyol Bul, 2021. 55(4): p. 465-479.

[19]. Aoki, S., et al., Transconjugation of erm(X) conferring high-level resistance of clindamycin for Cutibacterium acnes. J Med Microbiol, 2019. 68(1): p. 26-30.

[20]. Mercieca, L., et al., The Antibiotic Susceptibility Profile of Cutibacterium Acnes in Maltese Patients with Acne. J Clin Aesthet Dermatol, 2020. 13(6): p. 11-16.

[21]. Castellanos Lorduy, H.J., et al., Cutibacterium Acnes Tetracycline Resistance Profile in Patients with Acne Vulgaris, in a Colombian Dermatologic Center. Actas Dermosifiliogr (Engl Ed), 2021.

[22]. Yu, R., et al., Investigating Propionibacterium acnes antibiotic susceptibility and response to bacteriophage in vitro and in vivo. Front Microbiol, 2024. 15: p. 1424849.

[23]. Zhang, N., et al., Antimicrobial Susceptibility, Biotypes and Phylotypes of Clinical Cutibacterium (Formerly Propionibacterium) acnes Strains Isolated from Acne Patients: An Observational Study. Dermatol Ther (Heidelb), 2019. 9(4): p. 735-746.

| **Table S3 Basic information of each study** | | | | | | | | | | | | | | | | |
| --- | --- | --- | --- | --- | --- | --- | --- | --- | --- | --- | --- | --- | --- | --- | --- | --- |
|  |  |  |  |  |  | Number of antibiotic resistant isolated strains | | | | | | | | | | |
| First author  (year of publication) | Sample collection time | Regions | Number of isolated strains | Antimicrobial resistance criterion | Antibiotic drug sensitivity test method | Levofloxacin | Erythromycin | Roxithromycin | Azithromycin | Clarithromycin | Clindamycin | Tetracycline | Doxycycline | Minocycline | Chloramphenicol | TMP-SMX |
| Fan2016 | June to August 2014 | China (Chongqing,Sichuan, Yunnan,Shaanxi,Hubei) | 312 | CLSI | Agar dilution method | NA | 149 | NA | 149 | NA | NA | 2 | 1 | 0 | 1 | NA |
| Mendoza2013 | January 2005 to May 2006 | Colombia | 100 | NCCLS | Agar dilution method | NA | 35 | NA | NA | NA | 15 | 3 | 9 | 1 | NA | NA |
| Biswal2016 | 2010 to 2012 | India | 66 | CLSI | E-test | NA | 4 | NA | NA | NA | 0 | 4 | NA | 0 | NA | NA |
| Sheffer-Levi2020 | December 2017 to May 2018 | Israel | 36 | CLSI | E-test | NA | 9 | NA | NA | NA | 6 | 3 | 7 | 4 | NA | NA |
| Nakase2014 | 2009 to 2010 | Japan | 69 | CLSI | Agar dilution method | 3 | 16 | NA | NA | 16 | 13 | NA | 3 | 0 | NA | NA |
| Koyanagi2023 | 2019 to 2020 | Japan | 64 | CLSI | Agar dilution method | 7 | 27 | 27 | NA | NA | 27 | NA | 4 | 1 | NA | NA |
| Aoki2021 | 2013 to 2018 | Japan | 127 | CLSI | Agar dilution method | 10 | 65 | 65 | NA | 65 | 55 | NA | 4 | NA | NA | NA |
| Ishida2008 | 2006 to 2007 | Japan | 48 | CLSI | Agar dilution method | 0 | 5 | NA | NA | 5 | 4 | 0 | NA | 0 | 0 | NA |
| Song2011 | 2005.12-2007.12 | South Korea | 31 | EUCAST | E-test | NA | 0 | NA | NA | NA | 1 | 0 | 0 | 0 | NA | NA |
| Luk2013 | June to December 2009 | China (Hong Kong) | 86 | EUCAST | Agar dilution method | NA | 18 | NA | NA | NA | 46 | 14 | 14 | 14 | NA | NA |
| Schafer2013 | June 2008 to January 2009 | Chile | 80 | CLSI, EUCAST | Agar dilution method | NA | 10 | NA | NA | NA | 6 | 0 | 0 | NA | NA | 21 |
| Ma2016 | January 2015 to June 2015 | China (Shanghai) | 69 | CLSI | Agar dilution method | NA | 34 | NA | NA | NA | 23 | NA | NA | NA | NA | NA |
| Tang2018 | June 2015 to October 2016 | China (Guangdong) | 156 | CLSI | Agar dilution method | NA | 47 | NA | 76 | 131 | 45 | NA | NA | NA | NA | NA |
| Ma2021 | January to June 2019 | China (Shanghai) | 44 | CLSI | Agar dilution method | NA | 21 | NA | NA | NA | 25 | 0 | NA | NA | NA | NA |
| Sun2016 | June 2015 to February 2016 | China (Guangdong) | 70 | CLSI | Agar dilution method | NA | 23 | NA | 50 | 46 | NA | NA | NA | NA | NA | NA |
| Tang2024 | NA | China (Yunnan) | 139 | CLSI | E-test | NA | 59 | NA | NA | NA | 55 | 0 | 0 | 0 | NA | NA |
| Zeng2014 | 2009 to 2010 | China (Hubei) | 72 | NCCLS | E-test | NA | 19 | NA | NA | NA | NA | 0 | NA | NA | NA | NA |
| Özel Kaya2021 | NA | Turkey | 57 | CLSI, EUCAST | Agar dilution method | NA | 17 | NA | 20 | NA | 13 | 2 | 3 | NA | NA | NA |
| Aoki2019 | 2016 to 2017 | Japan | 34 | CLSI | Agar dilution method | NA | NA | NA | NA | NA | 15 | NA | NA | NA | NA | NA |
| Mercieca2020 | December 2015 to September 2017 | Malta | 100 | CLSI, EUCAST | E-test | NA | NA | NA | 18 | NA | 16 | 2 | 2 | 0 | NA | 0 |
| Castellanos Lorduy2021 | February 2017 to May 2018 | Colombia | 129 | CLSI | E-test | NA | NA | NA | NA | NA | NA | 7 | 7 | 1 | NA | NA |
| Yu2024 | August 2021 to October 2022 | China (Beijing) | 94 | CLSI | E-test | NA | 41 | NA | NA | NA | NA | NA | 0 | 0 | NA | NA |
| Zhang2019 | October 2016 to March 2017 | China (Shanghai) | 63 | CLSI | Agar dilution method | NA | 31 | NA | NA | NA | 18 | 0 | NA | 0 | NA | NA |
| NA,Not Available;EUCAST,European Committee on Antimicrobial Susceptibility Testing ;CLSI, Clinical and Laboratory Standards Institute;NCCLS,The National Committee for Clinical Laboratory Standards;E-test,Epsilometer test; | | | | | | | | | | | |  |  |  |  |  |

**Figure S1 Funnel plots and Egger test results for each study.**

**
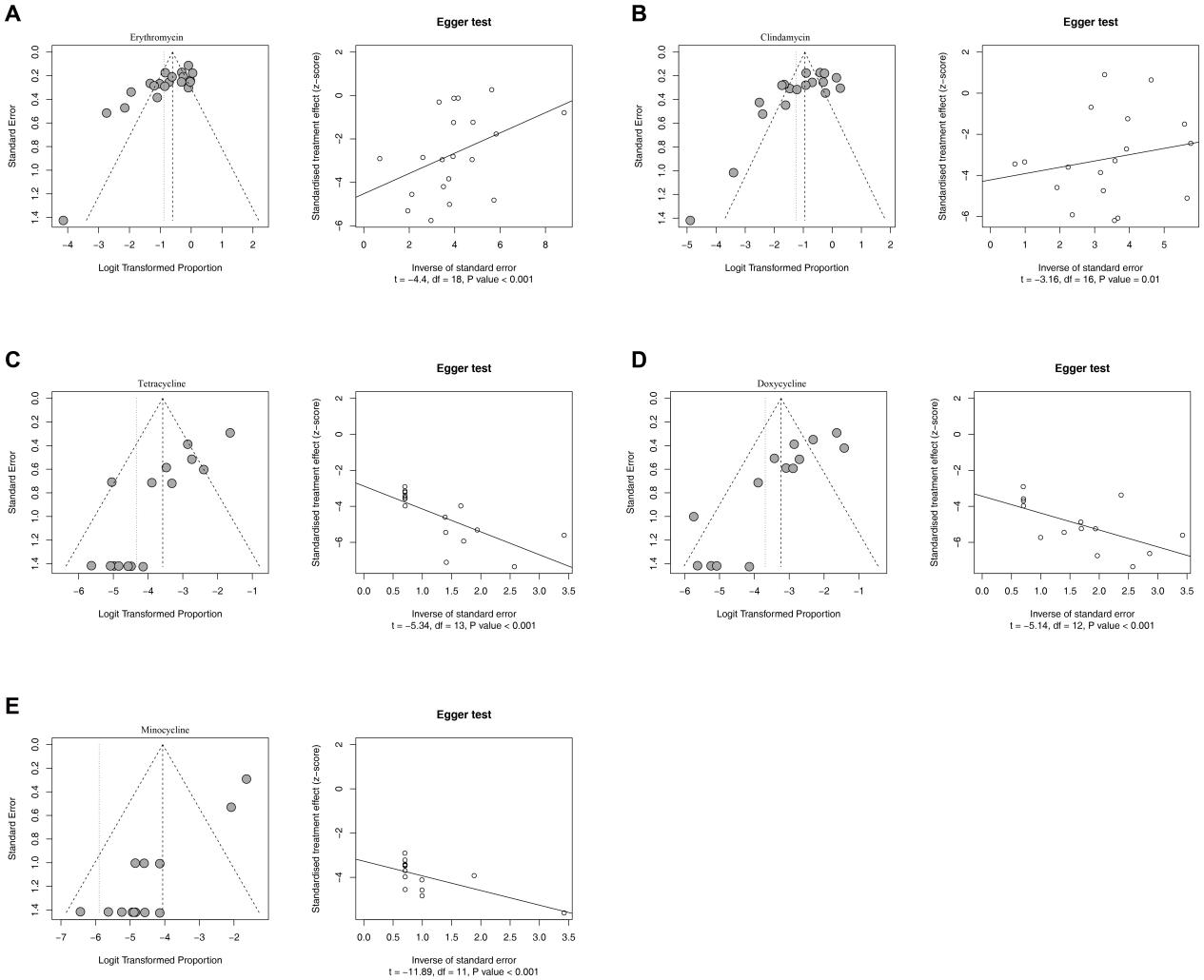
**

**Figure S2 Forest plots of macrolide resistance rates in *C. acnes*.**

**(A)** Forest plot of azithromycin resistance rates on different continents.

**(B)** Forest plot of erythromycin resistance rates on different continents.

**(C)** Forest plot of azithromycin resistance rates by different drug sensitivity test methods.

**(D)** Forest plot of erythromycin resistance rates by different drug sensitivity test methods.


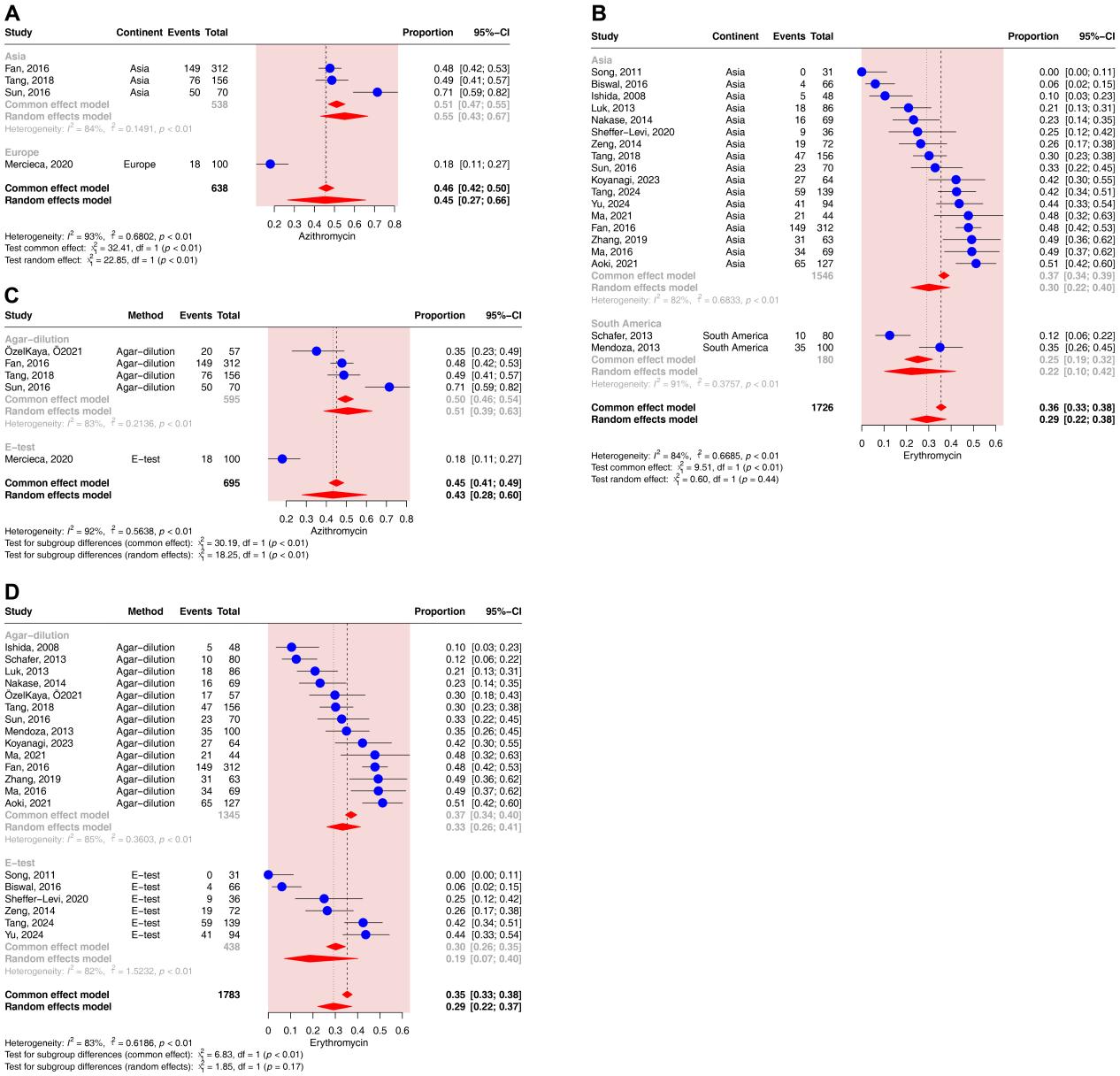


**Figure S3 Forest plots of lincosamide resistance rates in *C. acnes*.**

**(A)** Forest plot of clindamycin resistance rates on different continents.

**(B)** Forest plot of clindamycin resistance rates by different drug sensitivity test methods.


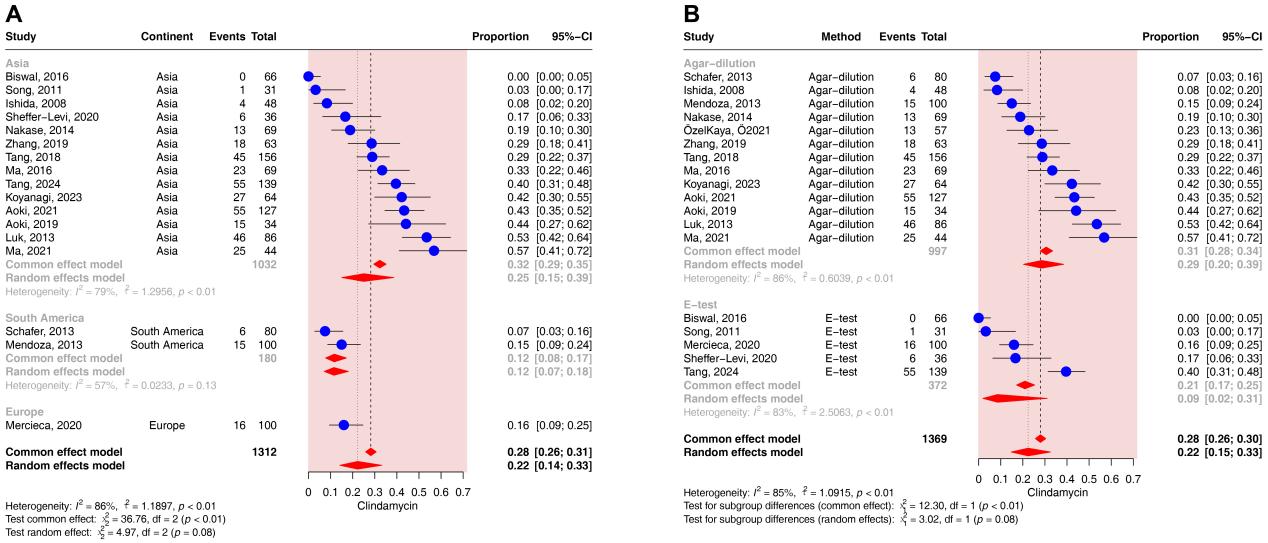


**Figure S4 Forest plots of tetracycline resistance rates in *C. acnes*.**

**(A)** Forest plot of doxycycline resistance rates on different continents.

**(B)** Forest plot of minocycline resistance rates on different continents.

**(C)** Forest plot of tetracycline resistance rates on different continents.

**(D)** Forest plot of doxycycline resistance rates by different drug sensitivity test methods.

**(E)** Forest plot of minocycline resistance rates by different drug sensitivity test methods.

**(F)** Forest plot of tetracycline resistance rates by different drug sensitivity test methods.

**
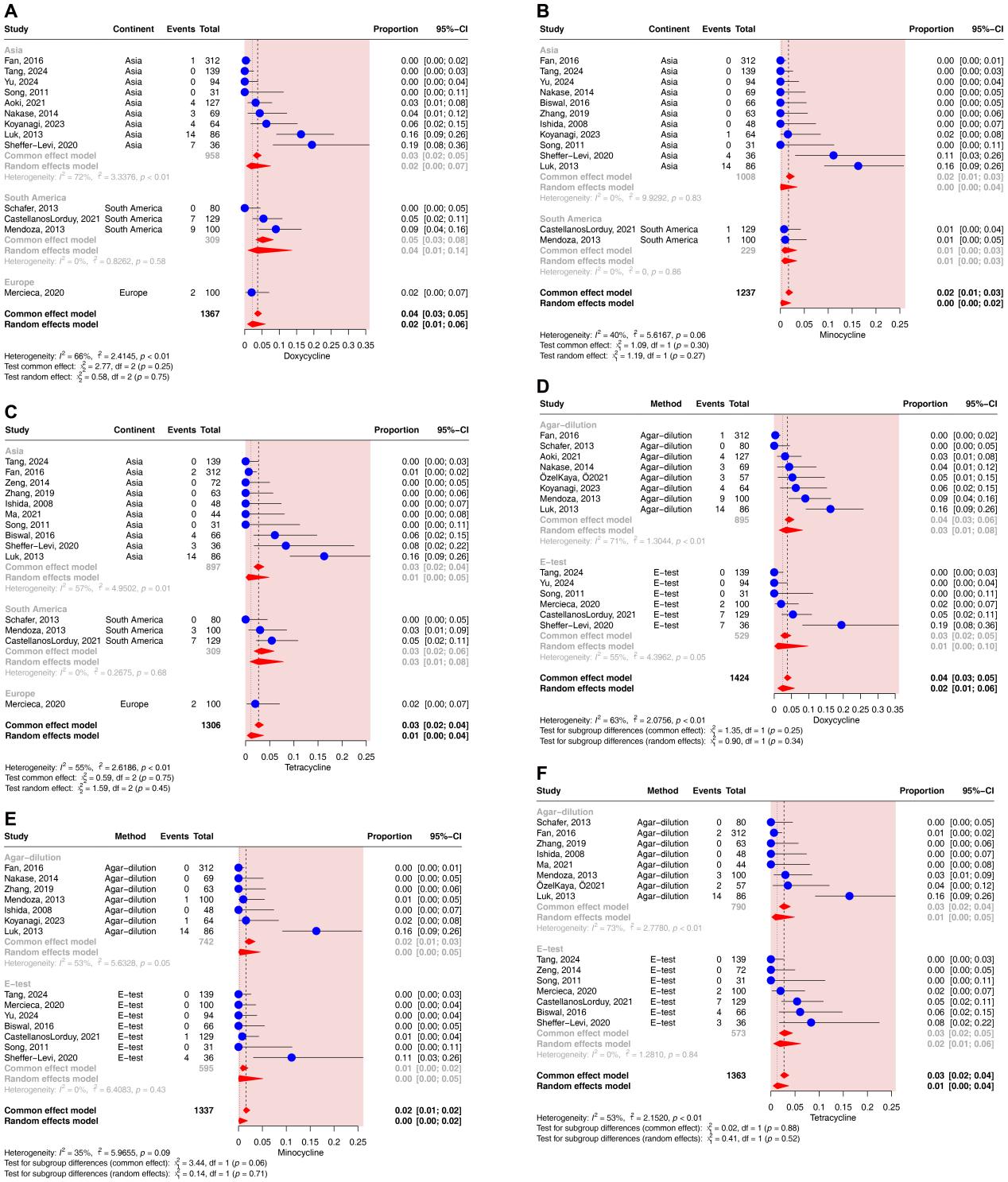
**

**Figure S5 Forest plots of chloramphenicol resistance rates in *C. acnes*.**

**(A)** Forest plot of chloramphenicol resistance rates in different countries.

**(B)** Forest plot of chloramphenicol resistance rates in different provinces of China.

**
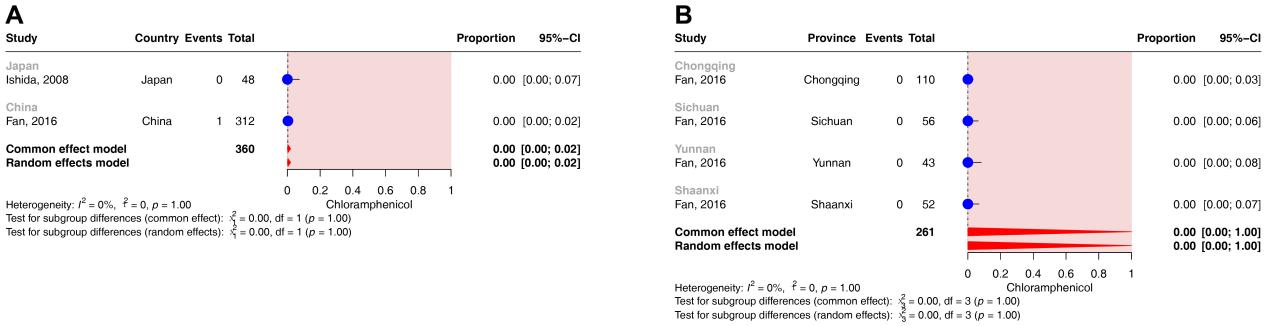
**

**Figure S6 Forest plots of TMP-SMX resistance rates in *C. acnes*.**

**(A)** Forest plot of TMP-SMX resistance rates in different countries.

**(B)** Forest plot of TMP-SMX resistance rates by different drug sensitivity test methods.

**
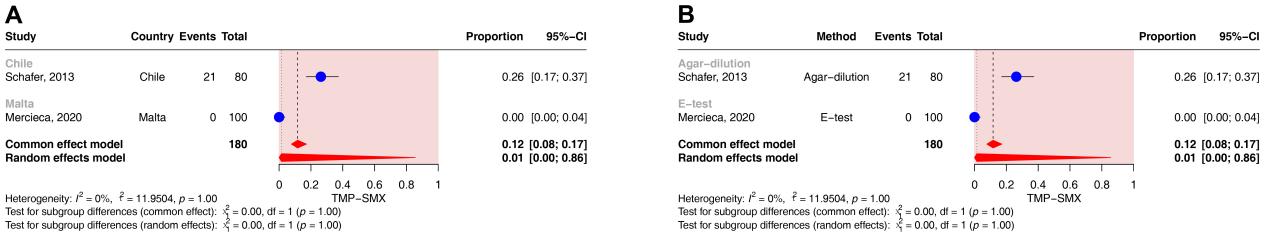
**
